# Fragmented Financing in Emergency Department Use Among US Veterans

**DOI:** 10.1001/jamahealthforum.2025.5635

**Published:** 2025-12-12

**Authors:** Anita A. Vashi, Tracy Urech, Siqi Wu, Steven Asch

**Affiliations:** 1 Center for Innovation to Implementation, VA Palo Alto Health Care System, Menlo Park, California; 2 Department of Emergency Medicine, University of California San Francisco; 3 Department of Emergency Medicine (Affiliated), Stanford University, Stanford, California; 4 Stanford Primary Care and Population Health, Stanford University, Stanford, California

## Abstract

This cross-sectional study uses US Veterans Affairs Health Care System administrative data crossed with New York state’s all claims database to identify emergency department visits by enrollees from 2016 to 2020.

## Introduction

The US Veterans Affairs (VA) Health Care System is recognized for its coordination and continuity of care, sometimes described as the VA advantage.^1^ Yet many veterans—particularly those who are dually or multiply insured—frequently seek care outside the VA network.^2^ Emergency department (ED) visits are a prime example: they often occur in time-sensitive situations where veterans present to the nearest facility, regardless of VA affiliation. These non-VA visits are commonly financed by Medicare, Medicaid, or private insurance and may never appear in VA records.

This fragmentation is increasingly consequential. The VA MISSION Act has expanded eligibility for VA-financed community care,^3^ while Medicare Advantage (MA) enrollment among veterans continues to rise.^4,5^ As a result, a growing share of veterans receive care in settings the VA neither finances nor documents—creating blind spots that impede follow-up, chronic disease management, and system oversight. Such fragmentation is inefficient, costly, and associated with worse outcomes.^6^

## Methods

To examine the scope of this issue, we linked VA administrative data with New York State’s all-payer claims database to identify ED visits by VA enrollees from 2016 to 2020. Data were analyzed from April 16 to June 27, 2025. The Stanford University institutional review board approved the cross-sectional study and waived informed consent per 45 CFR § 46.116(f)(3). This study followed the STROBE reporting guideline. We assessed the primary payer for each visit and categorized ED visits as financed by VA (direct or community care), traditional Medicare, MA, Medicaid, private insurance, self-pay/no charge, or other sources. The VA payment responsibility for non-VA ED visits varied based on coordination of benefits with other insurers (eMethods in Supplement 1). Follow-up was defined as any VA outpatient visit (primary, specialty, or mental health) within 30 days after the ED encounter; non-VA follow-up could not be observed. Analyses were performed using SAS Enterprise Guide version 8.3 (SAS Institute Inc).

## Results

**Figure 1.**
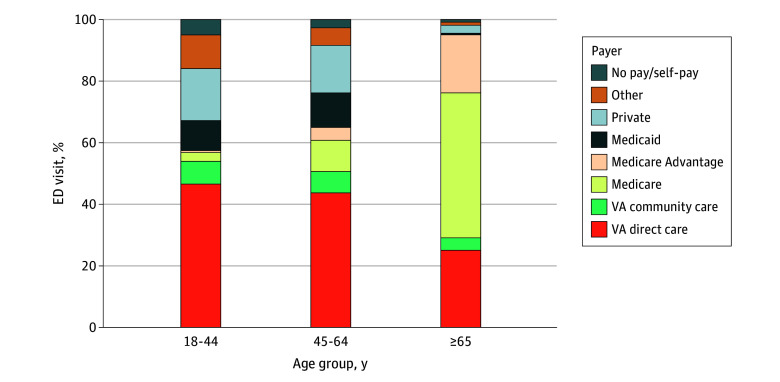
Distribution of Veterans’ Emergency Department (ED) Visits by Primary Payer and Age, 2016-2020 Veterans Affairs (VA) direct care is emergency care provided at VA emergency departments. VA community care is emergency care provided at a community (non-VA) hospital that is paid by VA. The Medicaid payer group includes Medicaid managed care. Other payers include other federal and local government programs. Department of Defense/Tricare accounted for 1.1% of encounters and is included in the Other payer category, along with Indian Health Service, correctional health systems, and workers’ compensation. The total number of ED visits by age group was 144 399 for enrollees aged 18 to 44 years; 304 541 for those aged 45 to 64 years; and 799 936 for those aged 65 years or older. Age was missing for 33 visits.

The study cohort included 1 248 909 ED visits by 293 689 unique VA enrollees residing in New York State. Most veteran ED care occurred beyond VA’s financial purview, with only 37.3% of visits financed by VA. The remainder were covered by Medicare (traditional and MA, 46.1%), private insurance (7.4%), Medicaid (4.2%), self-pay/no charge (1.8%), or other payers (3.3%) (Figure 1). Among the 799 936 ED visits (64.1%) made by veterans aged 65 years and older, Medicare was the dominant payer, financing 65.9% of ED visits, followed by the VA at 29.1%. In contrast, among ED visits made by veterans younger than 65 years, the VA was the top payer at 51.7%, followed by private insurance at 16.0%.

Geographic variation was modest but notable. Rural veterans were less likely to have VA-financed ED care overall with 30.5% vs 39.5% visits in urban areas but more likely to receive VA community care with 10.2% vs 3.4% visits.

**Figure 2.**
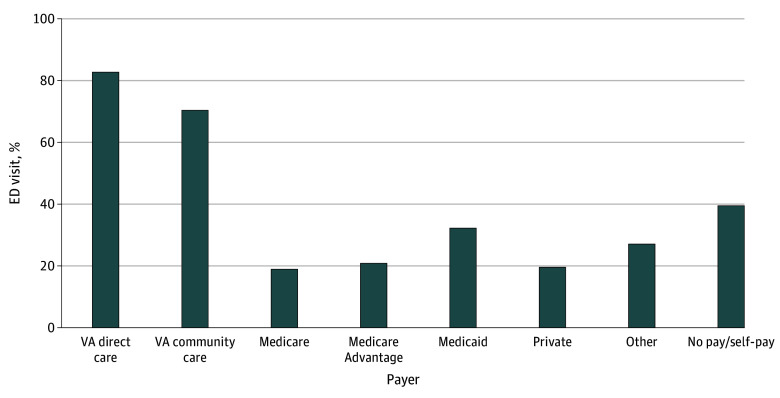
Percentage of Veterans Receiving Veterans Affairs (VA) Outpatient Follow-Up Care Within 30 Days by Primary Payer, 2016-2020 VA direct care is emergency care provided at VA emergency departments. VA community care is emergency care provided at a community (non-VA) hospital that is paid by VA. The Medicaid payer group includes Medicaid managed care. Other payers include other federal and local government programs (eg, Department of Defense/Tricare, Indian Health Service), corrections, and workers’ compensation. The total number of emergency department (ED) visits by payer category was 400 980 for VA direct care; 64 354 for VA community care; 411 310 for Medicare; 163 940 for Medicare Advantage; 52 050 for Medicaid; 92 710 for private; 40 746 for other; and 22 819 for no pay/self-pay.

VA follow-up rates varied substantially by payer (Figure 2). VA follow-up occurred after 82.7% of VA ED visits and 70.3% of VA community ED visits. In contrast, VA follow-up occurred in only 20.8% of the MA-financed visits, 18.9% of the traditional Medicare visits, and 19.5% of privately insured visits. Medicaid-financed visits had a slightly higher VA follow-up rate (32.2%). Follow-up rates also decreased with age, from 54.9% among veterans aged 45 to 64 years to 38.1% for those aged 65 years and older.

## Discussion

These findings reveal substantial fragmentation in emergency care for VA enrollees, based on the widespread presence of non-VA insurance coverage. Most ED visits are financed by non-VA payers and thus invisible to VA clinicians and care coordinators. This blind spot, compounded by the absence of requirements for non-VA clinicians to transmit clinical documentation, limits timely follow-up and management of complex patients. Even when the VA pays for care, lower follow-up rates after community ED visits suggest persistent coordination gaps. Limitations include the use of data from a single state, which may limit generalizability, and lack of information on non-VA follow-up care.

As the VA enrollee population ages, community care expands, and MA enrollment grows, the VA must strengthen benefits coordination and data-sharing infrastructure to safeguard Veteran care in an increasingly multipayer system.

## Notes

Supplement 1. **eMethods.** Coordination of Benefits Policy Context

Supplement 2.
Data Sharing Statement
