# Supplementary material for: Fragmented Financing in Emergency Department Use Among US Veterans
Source: JAMA Health Forum. 2025 Dec 12;6(12):e255635. doi: 10.1001/jamahealthforum.2025.5635 (PMC12701505; doi:10.1001/jamahealthforum.2025.5635)
Supplement: Supplement 1. — eMethods. Coordination of Benefits Policy Context [file jamahealthforum-e255635-s001.pdf]

Supplemental Online Content

Vashi AA, Urech T, Wu S, Asch S. Fragmented financing in emergency department use among US veterans. *JAMA Health Forum.* 2025;6(12):e255635. doi:10.1001/jamahealthforum.2025.5635

eMethods. Coordination of Benefits Policy Context

This supplemental material has been provided by the authors to give readers additional information about their work.

© 2025 Vashi AA et al. *JAMA Health Forum*.

eMethods. Coordination of Benefits Policy Context

VA has three legal authorities under which it can pay for emergency treatment at non-VA facilities:

1. **38 USC §1703 (Authorized Emergency Care):** Applied when a Veteran has a referral from a VA provider to an in-network community facility or when a Veteran self-presents to an in-network community facility for emergency care; notification is received timely (72 hours); and the Veteran meets defined eligibility criteria such as enrolled or exempt from enrollment in VA healthcare, VA not feasibly available and prudent layperson definition of emergency is met. Claims are generally reimbursed at 100% of Medicare rates.

2. **38 USC §1728 (Service-Connected Emergencies):** Applied to emergencies related to service-connected disabilities (or adjunct conditions), for Veterans permanently and totally disabled due to a service-connected condition, or during VA Vocational Rehabilitation. Claims are generally reimbursed at 100% of Medicare rates.

3. **38 USC §1725 (Non–Service-Connected Emergencies, Millennium Bill):** Applied to emergencies not related to service-connected disabilities. Claims are generally reimbursed at 70% of Medicare rates. VA often acted as a secondary payer, covering costs not paid by other health insurance (OHI), but historically not covering copayments or deductibles. This sometimes left Veterans with out-of-pocket liability.

Policy Shifts During and After the Study Period

- A 2016 court decision (*Staab v McDonald*) required VA to expand reimbursement obligations under §1725 to include situations where Veterans had Medicare or Medicaid coverage.
- Implementation of the MISSION Act (2019) and central contracting shifted most claims toward §1703.
- In 2019, 70% of non-VA emergency claims were processed under §1703, 18% were under §1725, and 11% were under §1728.
- By 2021, 94% of claims were processed under §1703.
- In January 2023, the COMPACT Act expanded VA authority to cover emergency suicidal crisis care at non-VA facilities, including ambulance transport, inpatient stabilization, and related outpatient services (such as follow-up visits, medications, and social work) for up to 90 days. This provision applies specifically to acute suicidal crises, not all emergency encounters.

© 2025 Vashi AA et al. *JAMA Health Forum*.

© 2025 Vashi AA et al. *JAMA Health Forum*.
