# Supplementary material for: Fragmented Financing in Emergency Department Use Among US Veterans
Source: JAMA Health Forum. 2025 Dec 12;6(12):e255635. doi: 10.1001/jamahealthforum.2025.5635 (PMC12701505; doi:10.1001/jamahealthforum.2025.5635)
Supplement: Supplement 2. — Data Sharing Statement [file jamahealthforum-e255635-s002.pdf]

Data Sharing Statement

Vashi. Fragmented Financing in Emergency Department Use Among US Veterans. *JAMA Health Forum*. Published December 12, 2025. doi:10.1001/jamahealthforum.2025.5635

Data
Data available: No

Additional Information
Explanation for why data not available: Due to US Department of Veterans Affairs (VA) regulations and our ethics agreements, the analytic data sets used for this study are not permitted to leave the VA firewall without a Data Use Agreement. This limitation is consistent with other studies based on VA data. However, VA data are made available to researchers with an approved VA study protocol.
